# Book Reviews

**Published:** 1874-02-01

**Authors:** 


### Book Reviews.

*Books received through Jansen, McClurg & Co., Chicago.*

THE Principles and Practice of Medical Jurisprudence. By Alfred Swaine Taylor, M.D., F.R.S., Fellow of Royal College of Physicians, Professor of Medical Jurisprudence and Chemistry in Guy's Hospital, etc. Second edition. Two vols. Philadelphia: Henry C. Lea, 1874.

This new edition of Dr. Taylor's well-known work on medical jurisprudence is one of the most complete and reliable treatises on this subject in our language. It is in two moderate-sized octavo volumes, bound in cloth. If you were to find any fault, it would be that some of the chapters are too diffuse, and enter into unnecessary details.

We wish, however, that every practitioner in America would procure this, or some other good work on the subject, and read it carefully. Almost every physician is liable, at some time, to be called into court as a witness; and nowhere else has our profession suffered more discredit in the estimation of the public than on the witness-stand.

In this work of Dr. Taylor's can be found not only the facts and laws pertaining to medical jurisprudence, but also all necessary rules for the guidance of practitioners in the investigation of cases and the giving of testimony. N. S. D.

---

A Handbook of the Theory and Practice of Medicine. By Frederick T. Roberts M.D., M.R.C.P., Fellow of the University College, Assistant Physician and Assistant Teacher of Clinical Medicine at University College Hospital, etc., etc. Philadelphia: Lindsay & Blakiston, 1874.

This is a neatly-printed volume of 1,052 pages, designed to contain a resume of practical medicine, in a concise and convenient form, for the use of students as a text-book. The author's style is clear, plain, concise, and methodical. He has overhauled all the topics usually included in works on practical and clinical medicine; and, though many of them are treated very briefly, yet, as a whole, the work presents a very fair digest of the prevalent views concerning special pathology and therapeutics. The publishers have not embellished it with any wood-cuts, or other illustrations. N. S. D.

---

Transactions of the Twenty-third Anniversary Meeting of the Illinois State Medical Society. Held at Bloomington May 20th and 21st, 1873. Chicago: Fergus Printing Company, 1873.

The volume of Transactions of the Annual Meeting of our State Medical Society, held last May, has finally made its appearance. It is a goodly volume of 268 pages, published in excellent style. Indeed, it has the best mechanical appearance of any volume heretofore published by the Society. It also embodies the first attempt to present, along with each paper, a report of the discusssions thereon. The effort appears to have been reasonably successful, and has certainly added much to the value of the work. We notice no less than *eleven* of those complimentary speeches — addresses of welcome, and replies thereto — which is rather too many, even if they were admitted to be good.

